# Sustained virological and biochemical responses to lamivudine and adefovir dipivoxil combination in a chronic hepatitis B infection despite mutations conferring resistance to both drugs

**DOI:** 10.1186/1476-5926-7-3

**Published:** 2008-03-12

**Authors:** Sylvie Larrat, Marie-Noëlle Hilleret, Raphaele Germi, Julien Lupo, Sandrine Nicod, Jean-Pierre Zarski, Jean-Marie Seigneurin, Patrice Morand

**Affiliations:** 1 Laboratoire de Virologie moléculaire et structurale, CHU de Grenoble BP 217, 38043 Grenoble cedex 09, France; 2 Département d'hépatogastroentérologie, CHU de Grenoble BP 217, 38043 Grenoble cedex 09, France

## Abstract

**Background:**

Sequential monotherapies of nucleotide analogs used in chronic hepatitis B treatment can lead to the selection of a resistance mutation to each antiviral drug.

**Case presentation:**

A patient with chronic hepatitis B was successively treated with lamivudine monotherapy, lamivudine-adefovir dual therapy, adefovir monotherapy and again with an adefovir-lamivudine dual therapy. Lamivudine-associated mutations (rtL180M and rtM204V/I) followed by adefovir-associated mutations (rtN236T and rtA181V) emerged during the two monotherapy regimens. Despite the presence of rtM204V/I, rtA181V, and rtN236T mutations at the beginning of the second dual therapy, sustained biochemical and virological responses have been observed thus far after 23 months.

**Conclusion:**

This case illustrates that rtM204V/I, rtA181V, and rtN236T resistance mutations can coexist in a patient but do not preclude the recycling of lamivudine and adefovir in combination therapy, when no other therapeutic choices are available.

## Background

The treatment of chronic hepatitis B with oral nucleoside (e.g., lamivudine, entecavir) and nucleotide (e.g., adefovir) analogs that inhibit viral polymerase reverse transcriptase activity has dramatically modified the management of infected patients but is hampered by the emergence of resistant strains containing mutation in the reverse transcriptase (rt) part of the HBV polymerase gene (HBV Pol). The main lamivudine resistance mutations were mapped in the C and B domains of HBV Pol, and the specific mutations selected were rtM204I/V/S (domain C) and rtL180M (domain B) [1]. Resistance to adefovir is associated with B and D domain enzyme mutations. The major mutations observed with adefovir-resistant HBV are identified as rtN236T (domain D) and rtA181V (domain B) [2-5]. Lamivudine-resistance mutations were detected in 15–30% of treated patients after 1 year of therapy and up to 70% after 5 years [6]. Resistance to adefovir is thought to be less common and occurs later in the course of treatment as compared to lamivudine [7,8]. Nevertheless, a rate of 29% has been described after 5 years of therapy [9].

## Case presentation

**Figure 1:**
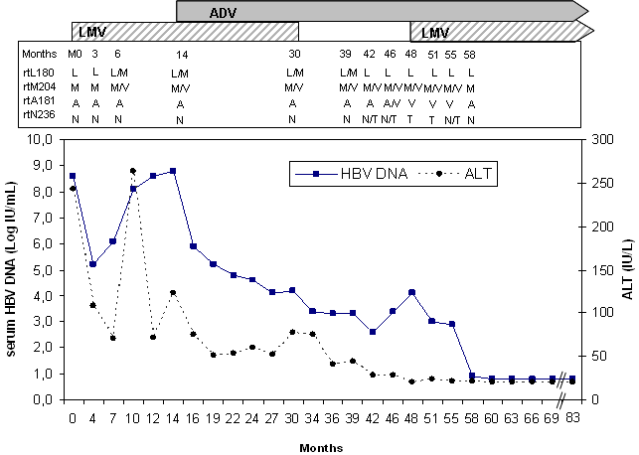
**HBV viral load and alanine aminotransferases (ALT)**. HBV viral load and ALT from a patient with chronic hepatitis B during the course of antiviral therapy. HBV polymerase gene mutations detected by sequencing and line probe assay are displayed in the panel above the graph.

In 2000, a 35-year-old Turkish man (91 kg for 1.70 m; BMI: 31.5) was found to have an abnormal level of serum alanine aminotransferase (ALT = 243 IU/L; normal, < 42 IU/L) after undergoing routine blood testing. The patient was HBs-antigen (Ag)-positive, HBs-antibody (Ab)-negative, HBe-Ag-negative, HBe-Ab-positive, and HBc-Ab-positive (Axsym, Abbott Laboratories, North Chicago, IL, USA). Hepatitis B virus (HBV) DNA was detected as positive in the serum. Serological markers for hepatitis delta virus, human immunodeficiency virus, and hepatitis C virus were negative. The persistence of biochemical and virological abnormalities was an indication for a liver biopsy, which showed chronic hepatitis B with a mild necroinflammatory activity and moderate fibrosis (Metavir score, A1F2). Treatment with lamivudine 100 mg/day was initiated in November 2000 (month 0: M0). At that time, serum HBV DNA load was 8.6 log_10 _IU/mL (Amplicor-HBV-Monitor, Roche Diagnostics, Meylan, France; limit of detection: 200 copies/mL) (Figure 1). After an initial 3.4 log_10 _drop of the HBV DNA load within the first 3 months of therapy, the serum HBV DNA load regularly increased with a flare of ALT at M10 and reached the pretreatment level at M14. Consequently, adefovir 10 mg/day was added to the ongoing lamivudine therapy in a temporary-use authorization program. This dual-therapy regimen was maintained for 15 months with a good virological and biochemical response (HBV DNA load: 4.1 log_10_IU/mL and ALT = 41 IU/L), leading to lamivudine interruption at M30 because, at that time, the benefit of maintaining dual therapy was not proven. With adefovir monotherapy, the serum HBV DNA level continued to decrease with a nadir at M42 (2.6 log_10_IU/mL). However, a virological breakthrough (i.e., a controlled increase in serum HBV load > 1 log) occurred at M48, leading to the re-introduction of lamivudine in association with adefovir (at that time entecavir was not yet available). With this second dual therapy, serum HBV DNA became undetectable at M60 (COBAS Taqman HBV, Roche Diagnostics, Meylan, France; limit of detection: 6 IU/mL). To date (M83), the HBV load is still undetectable and ALT levels have been repeatedly within the normal range.

In order to detect lamivudine and adefovir resistance-associated mutations and to determine the HBV genotype of the patient strain, sequence analyses were performed within the viral polymerase and the precore genes using a bi-directional sequencing with Ceq 2000 XL Beckman (Beckman-Coulter, Villepinte, France). The presence of resistance-associated mutations was also assessed using a commercially available reverse hybridization line probe assay (InnoLipa-DR2-assay, InnoGenetics, Ghent, Belgium), following the manufacturer's instructions. This second genotyping assay was performed because of its sensitivity in detecting HBV resistance-associated mutations, particularly in low HBV-load samples [10,11]. To evaluate the proportion of mutated strain in each sample, we used an in-house selective real-time PCR, which provides a quantitative detection of the main HBV mutations associated with lamivudine and adefovir resistance: rtM204V/I and rtN236T, respectively. This method was derived from the strategy called the amplification refractory mutation system (ARMS) [12]. Briefly, discrimination between wild type and mutant with a single base pair mismatch is made possible using a primer-template mismatch at the 3' end of the primer, which significantly compromises polymerase efficiency in amplifying the wild type strain. Primers and probes for rtM204V/I were described previously and the assay can detect up to 0.1% variants for a total viral load of 10^5 ^copies/mL [13]. We designed and evaluated a specific reverse primer (5'-ATCTTTTTGTTTTGTTAGGGG-3') for the detection of the rtN236T-mutation.

The phylogenetic analysis of the HBsAg-coding region sequence showed a genotype D and a stop codon mutation at the G1896A position in the precore region, confirming the serological data for the infection with a precore mutant HBV strain (data not shown).

The viral polymerase gene sequencing before and after the first 3 months of lamivudine monotherapy revealed a wild viral strain. The two lamivudine-resistance mutations rtL180M and rtM204V/I were concomitantly detected with sequencing and line probe assays from M6 to M39 (9 months after cessation of lamivudine). During this period, the rate of rtM204V/I strain remained stable, as shown by the ARMS assay (see Table 1). After M39, the rtL180M variant was no longer detectable with sequencing or the line probe assay. From M42 to M55, the rtM204V/I variant was still detectable with ARMS and the line probe assay but not with sequencing because the mutated population represents less than 25% of the total viral population, as seen by ARMS (see Table 1).

**Table 1:** Mutated viral strain analysis. Percentages of viral strain carrying the rtM204V/I or the rtN236T mutation determined with in-house selective real-time PCR (ARMS).

Date	Treatment	HBV viral load (log IU/mL)	% rtM204 (wt)	% rtM204V	% rtM204I	% rtN236 (wt)	% rtN236T
M0	LMV	8.6	100	0	0	-	-
M3	LMV	5.2	100	0	0	-	-
M7	LMV	6.1	28	48	24	-	-
M10	LMV	8.1	34	45	21	-	-
M12	LMV	8.6	22	58	20	-	-
M14	LMV	8.8	30	50	20	-	-
M16	LMV + ADV	5.9	8	67	25	-	-
M19	LMV + ADV	5.2	37	43	20	-	-
M22	LMV + ADV	4.8	24	51	25	-	-
M24	LMV + ADV	4.6	26	49	25	-	-
M27	LMV + ADV	4.1	66	19	15	-	-
M30	LMV + ADV	4.2	66	22	12	-	-
M36	ADV	3.3	83	7	10	100	0
M39	ADV	3.3	74	8	18	100	0
M42	ADV	2.6	75	8	17	96	4
M46	ADV	3.4	82	8	10	79	21
M48	ADV	4.1	98	2	0	66	34
M51	LMV + ADV	3.0	87	0	13	71	29
M55	LMV + ADV	2.9	86	14	0	73	27

Note: wt = wild type, -: not done

The first adefovir-resistance mutation rtN236T has been observed since M42 (after 28 months of adefovir therapy, including 12 months of adefovir monotherapy) using ARMS and the line probe assay. The second adefovir-resistance mutation rtA181V was detected at M46.

Thus, at the beginning of the second-line dual therapy (M48), rtA181V, rtN236T, and rtM204V/I variants were detectable either by sequencing analysis or both the line probe assay and ARMS. This resistance profile persisted at M55 but the rtN236T-mutated strain proportion slightly decreased (from 34% at M48 to 27% at M55). The line probe assay carried out at M58 showed wild type recovery of amino acids 204, 236, and 181. At this time, viral load was 8 IU/mL and sequencing and ARMS detection were unsuccessful. Finally, the viral load became undetectable (< 6 IU/mL with Cobas-TaqMan-HBV) at M60 after 1 year of dual therapy.

## Discussion

In this report, we describe a patient with chronic hepatitis B who achieved a sustained virological and biochemical response to a second-line of combination therapy with lamivudine and adefovir despite the presence of rtM204V/I, rtN236T, and rtA181V mutations. This response has been maintained so far and the three mutations were no longer detectable, even with sensitive genotyping assays, after 10 months of this dual therapy.

To our knowledge, cases of similar combined mutations are rare. Villet *et al*. [14] reported a patient who, on the same viral strain, showed a combination of rtV173L, rtL180M, rtA181T, and rtN236T mutations after several successive courses of lamivudine and adefovir. In this case, the rtM204V/I mutation was not detectable. More recently, Karatayli *et al*. [15] described a patient who did not respond to adefovir and lamivudine. They demonstrated the presence of rtM204I and rtA181S mutations in the HBV polymerase and the phenotypical resistance of this strain to both adefovir and lamivudine. Thus, the concomitant association of rtM204V/I/S and rtN236T is very rare and has even been described as mutually exclusive by Osiowy *et al*. [16].

The successful addition of lamivudine to the adefovir regimen in spite of the presence of rtN236T-mutation has been reported *in vivo *[2,5], but in these cases no previous lamivudine resistance-associated mutation was detectable. Moreover, when the rtA181V mutation is present in combination with the rtN236T mutation, lamivudine is no longer recommended. Indeed, the rtA181V mutation has been responsible for the reduction of lamivudine susceptibility both *in vitro *and *in vivo *[17].

Furthermore, Brunelle *et al*. [18] determined the phenotypical characteristics of an HBV-laboratory strain carrying rtL180M, rtM204V, and rtN236T. This mutant was approximately 59-fold less susceptible to lamivudine and adefovir-combination than wild type HBV. Comparatively, this mutant was approximately four- and six-fold less susceptible to tenofovir and entecavir therapy than wild type HBV. This correlates with the clinical setting in which successful rescues with tenofovir or entecavir against adefovir-resistant HBV have been reported [3,4,19].

It should be noted that the antiviral response during the first course of dual therapy was only slow and partial, whereas the second course led to a rapid and complete viral response. The reasons for this difference are not clear and could stem from several intricate factors such as variability in the patient's compliance and/or differences in the antiviral efficacy of the added drug against the resistant strain. The explanation of the sustained response to the second dual therapy in spite of the presence of the rtM204V/I, rtA181V, and rtN236T mutations also remains unclear. One explanation could be that the rt204 and rt236 mutations are not present on the same genome and that the low level of rt204 mutant observed with the ARMS assay at the beginning of the dual therapy did not prevent the biochemical and virological response. The different percentages of mutated strains rtM204V/I and rtN236T observed with the ARMS assay argue in favor of this hypothesis, but the significance of these low percentages is questionable. Thus, only clonal sequencing analysis could confirm the presence of these two mutations on different strains [20].

## Conclusion

This case illustrates that rtM204V/I, rtA181V, and rtN236T resistance mutations can coexist in a patient. We conclude that given an unfavorable resistance profile this case suggests the possibility, when no other treatment option is open, of recycling previously used drugs in combination therapy.

## Competing interests

The author(s) declare that they have no competing interest.

## Authors' contributions

JL and SN carried out the virological analyses. SL interpreted the data and wrote the manuscript. MNH provided clinical care for the patient and analyzed clinical data. PM and RG helped to interpret the data and participated in the writing of the manuscript. JMS and JPZ critically revised the manuscript.

## Consent

Written informed consent was obtained from the patient for publication of this case report.
